# IMPLEMENTING ISUPPORT IN UGANDA: A PILOT RCT FOR REDUCING CAREGIVER STRESS

**DOI:** 10.1093/geroni/igae098.0187

**Published:** 2024-12-31

**Authors:** Joy Louise Gumikiriza, Kamada Lwere

**Affiliations:** Makerere University, Kampala, Kampala, Uganda; Makerere University, Kampala, Kampala, Uganda

## Abstract

Background: Dementia is a significant public health issue in low- and middle-income countries, and Uganda has seen a notable increase in cases. The iSUPPORT program, adapted into AiSUPPORT, provides tailored interventions that address the sociocultural and economic context of Uganda, aiming to alleviate psychological distress among caregivers of patients with AD and dementia. Methods: This pilot randomized controlled trial (RCT) was conducted in the Wakiso District, enrolling 65 caregivers of Alzheimer’s disease and related dementia (ADRD) patients. Participants were randomly assigned to either an intervention group that received AiSUPPORT sessions, or a control group that received standard care. This study aimed to assess the feasibility, fidelity, acceptability, and impact of the intervention on caregivers’ psychological distress, quality of life, and depression severity, as measured using the Kessler Psychological Distress Scale and other tools. Results: In the intervention group, psychological distress scores reduced significantly, averaging 29.2 to 23.7 (p< 0.001), and depression severity scores dropped from 33.4 to 25.6 (p< 0.001). Quality of life scores improved substantially, from an average of 81.1 to 89.4 (p< 0.001), compared with the control group. The AiSUPPORT program demonstrated effectiveness and cultural appropriateness, with a 95% retention rate and high engagement. Conclusion: The AiSUPPORT program for dementia caregivers in Uganda’s successful implementation in low-resource settings demonstrates its potential. The program’s effectiveness, feasibility, and cultural sensitivity underscore the importance of cultural considerations in intervention design, supporting the integration of culturally adapted evidence-based interventions in national health systems to address the complex needs of dementia caregivers.

